# Reaction of Lactone-Containing Poly(benzofuran-*co*-arylacetic acid) with Diamines to Cross-Linked Products of Improved Thermal Conductivity

**DOI:** 10.3390/molecules29246020

**Published:** 2024-12-20

**Authors:** Alexandrina Nan, Xenia Filip, Jürgen Liebscher

**Affiliations:** 1 National Institute for Research and Development of Isotopic and Molecular Technologies, Str. Donat 67-103, 400293 Cluj-Napoca, Romania; alexandrina.nan@itim-cj.ro (A.N.); filip.xenia@itim-cj.ro (X.F.); 2 Institute of Chemistry, Humboldt-University of Berlin, 12489 Berlin, Germany

**Keywords:** poly(benzofuran-*co*-arylacetic), phenoplast, lactone, diamines, amide formation, cross-linking reaction, thermal conductivity, thermal diffusivity

## Abstract

The recently developed phenoplast-related polymer, poly(benzofuran-*co*-arylacetic acid), presents a versatile molecular structure containing lactone and carboxylic acid functionalities that offer significant flexibility in creating cured materials with tailored properties for diverse applications, wherein also the thermal conductivity is an important factor. This study analyses the possibility of forming amide moieties of poly(benzofuran-*co*-arylacetic acid) with diamines resulting in cross-linked products in order to control its thermal properties. The cross-linking process is achieved by utilizing three distinct diamines, 1,6-diaminohexane, *p*-xylylenediamine, and 4,7,10-trioxa-1,13-tridecanediamine, each possessing different degrees of polarity, flexibility, and reactivity. The resulting cross-linked zwitterionic poly(benzofuran-*co*-arylacetic acids) were structurally and morphologically characterized. By means of measuring the thermal conductivity and diffusivity of the materials, the possibility of adjusting the thermal properties of the cross-linked products by choosing appropriate linkers was determined. A case was developed where the thermal conductivity and diffusivity increased with temperature, a hardly found property in the cross-linking of polymers being important for many practical applications.

## 1. Introduction

Substituting metals with polymers in heat exchanger applications can present significant obstacles. These difficulties include issues such as poor mechanical strength, high thermal expansion, physical and environmental ageing, limited temperature capacities, and challenges in joining components. Furthermore, common polymers are thermal insulators and have low thermal conductivities on the order of 0.1 W/(mK), making them less than optimal for heat exchangers [1,2,3,4,5]. Developing a polymer that exhibits high thermal conductivity would enable the production of polymer heat spreaders and heat exchangers with exceptional characteristics. These include structural compactness, lightweight, corrosion resistance, ease of processing, and low cost, thereby opening a wide range of potential applications in electronics, water, and energy industries [6,7]. Thus, enhancing the thermal conductivity of polymers and polymer composites is a subject of great interest. Over the last two decades, a better understanding of the underlying process of heat transfer at micro, nano, and even molecular scales has led to significant efforts to improve the thermal conductivity of polymers and polymer nanocomposites to enable a broader range of applications [8,9,10,11,12,13,14,15]. It turned out that the morphology of a polymer plays a crucial role in determining its thermal conductivity. In the context of bulk polymers, disordered chains and random defects, such as chain entanglements and chain ends, are known to result in low thermal conductivity. When the amorphous domains are dominant, the vibrational modes in the polymer tend to be localised, resulting in low thermal conductivity [3]. Therefore, it can be expected that the thermal conductivity can be increased by aligning the polymer chains more effectively. Indeed, various methods, such as mechanical stretching, cross-linking reactions, nanoscale patterning, and electrospinning, have been employed to obtain polymers with well-aligned chains for high thermal conductivity [16,17,18,19,20,21].

Cross-linking is of great importance in polymer chemistry because it considerably alters the polymer properties such as stiffness, density, solubility, thermal behaviour, and chemical stability, thus allowing them to be adapted for practical application. Prominent examples are the cross-linking of latex applied in rubber industries and the generation of cross-linked polyacrylamide useful for soft contact lenses. The process of cross-linking connects different polymer chains either by covalent bonds or ion-ion interactions. It can be implemented in two principal ways: by reaction of a polymer with the cross-linking agent or by synthesising the polymer starting from monomers containing functional groups capable of cross-linking either during the polymerisation or by subsequent heating [22,23]. Cross-linking polymer chains via strong covalent bonds can improve the efficiency of heat conduction pathways and networks in polymers. It is a reasonable assumption that the polymer’s thermal conductivity increases with the number of cross-links formed. Tonfeng et al. (2011) have demonstrated that increasing the cross-linking density of polyethylene results in a 50% improvement in thermal conductivity [24]. Furthermore, molecular dynamics simulations have indicated that cross-linking in polyethylene can significantly increase its thermal conductivity, proportional to the cross-linking density [25]. These findings suggest that cross-linking can be an effective strategy for enhancing the thermal properties of polymers, particularly in applications where heat transfer is critical.

The present study aims to shed light on the reaction of poly(benzofuran-*co*-arylacetic acid) (**PBAAA**), a somehow phenoplast-related polymer, with various aliphatic and aromatic diamines, resulting in efficient cross-linkage. Diamines were chosen to differ in polarity and stiffness in order to find optimal structural properties. Spectroscopic techniques were employed to characterise the resulting cross-linked **PBAAA**. The study mainly focuses on exploring the mode of amide formation and the influence of diamine as a cross-linking agent on the thermal conductivity of **PBAAA**.

## 2. Results and Discussion

### 2.1. Synthesis of Cross-Linked PBAA with Diamine

**Scheme 1:**
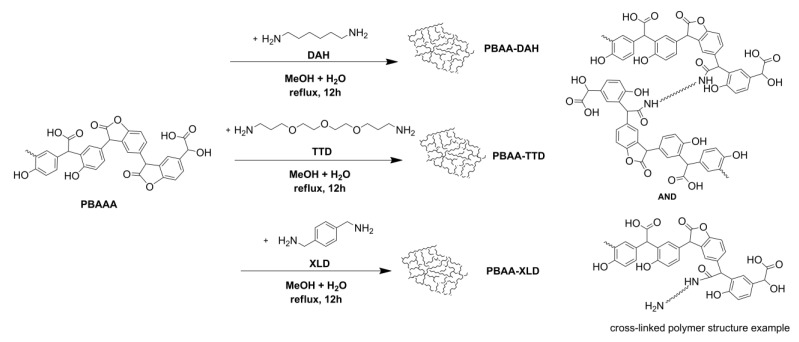
Preparation of cross-linked PBAAA with different diamines.

As part of our interest in developing new polymers with abundant functional groups, we carried out the controlled thermal polycondensation of 4-hydroxymandelic acid. Multiple Friedel Crafts alkylation takes place connecting the phenolic moieties, yielding polymer chains resembling somewhat the well-known phenoplast but are not cross-linked and contain, in addition to the phenolic hydroxy groups, carboxyl and lactone functionalities as part of a benzofurane ring. Due to these moieties, the resulting poly(benzofuran-*co*-arylacetic acid) (**PBAAA**) provides ample possibilities for derivatisation, including cross-linkage by bifunctional reagents (Scheme 1). Cross-linking of **PBAAA** with diamines was conducted with methanol/water in a straightforward manner by reflux for 24 h. The cross-linked polymers **PBAA-DAH**, **PBAA-TTD**, and **PBAA-XLD** are solids that were separated via filtration, subjected to multiple washings with water and methanol, and subsequently characterised. The three resultant cross-linked polymers exhibit distinct colourations: PBAA-DAH is characterised by a dark brown hue, PBAA-TTD presents a yellow-brown tone, and PBAA-XLD reveals a muted pink colour (see Figure S1). It is important to note that the synthesis process begins with a burgundy-purple polymer.

The cross-linking of the **PBAAA** polymer can occur by amide formation of the carboxyl and lactone groups, while the latter is the more reactive moiety and is ring-opened. When different polymer chains are attacked, this results in cross-linking, which is crucial for affecting the thermal properties. Although the carboxyl function is less reactive, it can also form amides under the applied reflux conditions. Thus, the cross-linking of **PBAAA** chains can take place between two lactone rings, two carboxyl groups, or a lactone ring and a carboxyl group. The resulting amide moieties are the same, regardless of which functional groups they are formed. Scheme 1 outlines the cross-linking reaction of **PBAAA** with various diamines, specifically **DAH**, **TTD**, and **XLD**.

### 2.2. ss-NMR of the Cross-Linked PBAAA

To illustrate the chemical structure of the cross-linked polymers, we commenced our analysis by scrutinising the ^13^C-NMR and ^15^N-NMR spectra of the solid cross-linked polymers.

**Figure 1:**
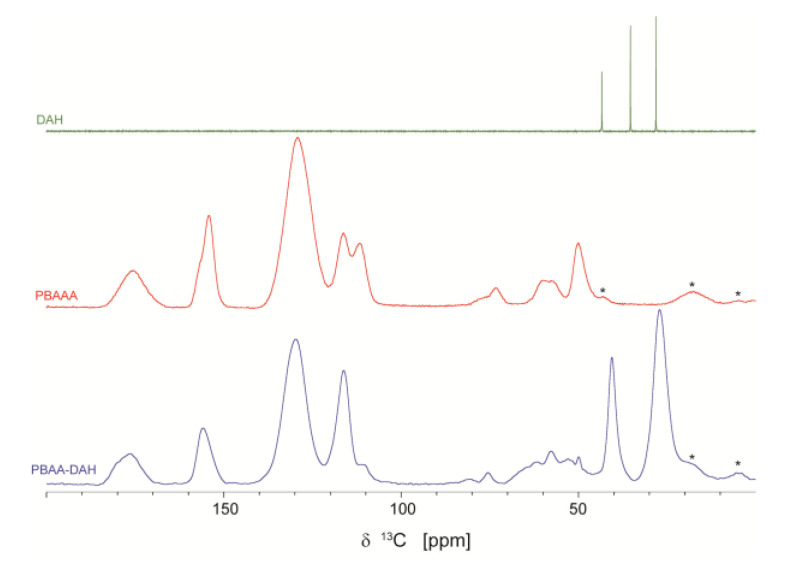
The ^13^C ss-NMR spectra of **PBAAA** (red), **PBAA-DAH** (blue), and **DAH** (green) recorded by the CP-MAS sequence as described in Section 3; asterisks * indicate sidebands.

Significant changes were observed in the ^13^C *ss*-NMR spectrum of **PBAA-DAH** with respect to precursor **PBAAA** (Figure 1).

**Scheme 2:**
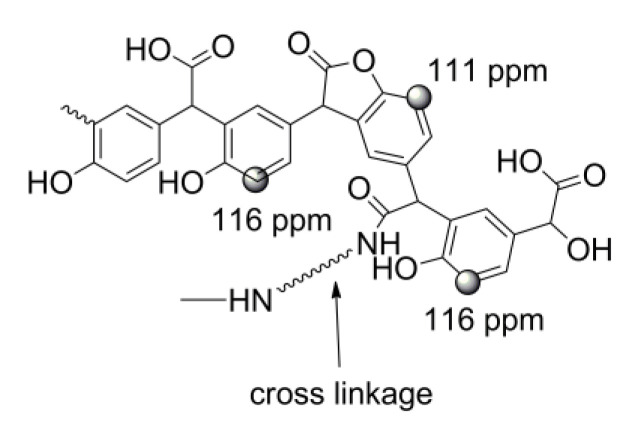
Carbon atoms with altered ^13^C NMR shifts after the cross-linking reaction.

The cross-linking reaction was confirmed by the presence of the new peaks at 28 ppm and 40 ppm. These peaks were ascribed to the -CH_2_ groups present in the cross-linking molecule **DAH**. The **PBAA-DAH** spectral analysis demonstrates a discernible peak at 50 ppm corresponding to the -CH groups from the lactonic ring. However, this peak appears much more feeble than that observed in the ^13^C *ss*-NMR spectrum of **PBAAA**. This observation suggests that a significant portion of the lactone ring is opened during the cross-linking reaction. As another indication of successful ring opening of the lactone, the signal at around 111 ppm, typical for the benzofurane ring, diminished while the 116 ppm signal of the phenol increased considerably (Scheme 2).

**Figure 2:**
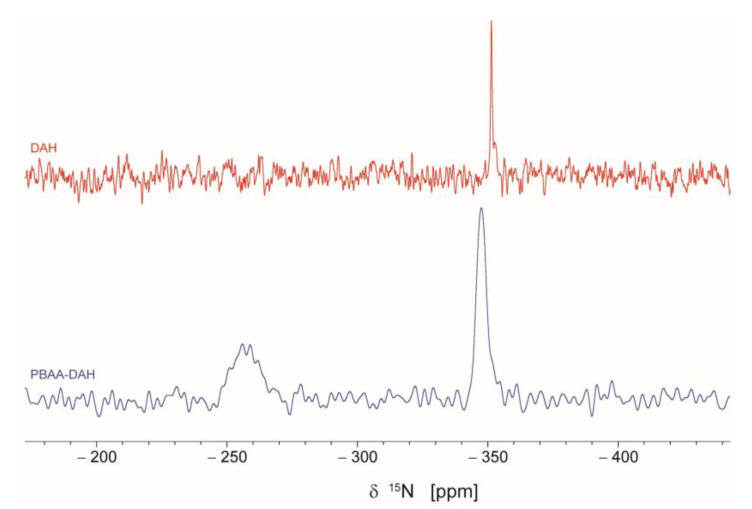
The ^15^N ss-NMR spectra of **PBAA-DAH** (blue) and **DAH** (red) recorded by the CP-MAS sequence as described in Section 3.

The detection of free diamine in the polymer through ^13^C *ss*-NMR is unattainable. Instead, ^15^N *ss*-NMR spectra of the cross-linked polymer **PBAA-DAH** and the cross-linking agent **DAH** were utilised to detect if some unreacted diamine was present in the cross-linked polymer matrix. Figure 2 presents a comparison of these two spectra. Two peaks are discernible in the ^15^N *ss*-NMR spectrum of the cross-linked polymer **PBAA-DAH**. The peak at −255 ppm is characteristic of the nitrogen atom involved in forming the new amide bond in the cross-linking reaction. The peak at −345 ppm corresponds to an amino nitrogen atom (-NH_2_). This suggests that some of the **DAH** molecules do not form amides with both amino groups but only with one, while the other remains untouched. Because the ^15^N atom signal in the **PBAA-DAH** spectrum at −345 ppm is much broader and shifted compared to **DAH**, it cannot be caused by unreacted **DAH** remaining adsorbed in the product. Indicating that only one end of the cross-linking agent remains free, with the other being bound. In conclusion, the polymer **PBAA-DAH** contains cross-linking amide and non-cross-linking amide moieties, the latter containing a primary amino group, giving rise to zwitterion formation with carboxyl groups. Free amino groups are also present in the other two cross-linked polymers, **PBAA-TTD** and **PBAA-XLD**, as demonstrated with the aid of ^15^N *ss*-NMR spectra (Figures S2 and S3). The ^15^N *ss*-NMR spectrum of **XLD** displayed a signal at −370 ppm, which was considerably broader than the peak present in the ^15^N *ss*-NMR spectrum of **PBAA-XLD** (Figure S3). Moreover, this signal was shifted to lower values at −342 ppm.

**Figure 3:**
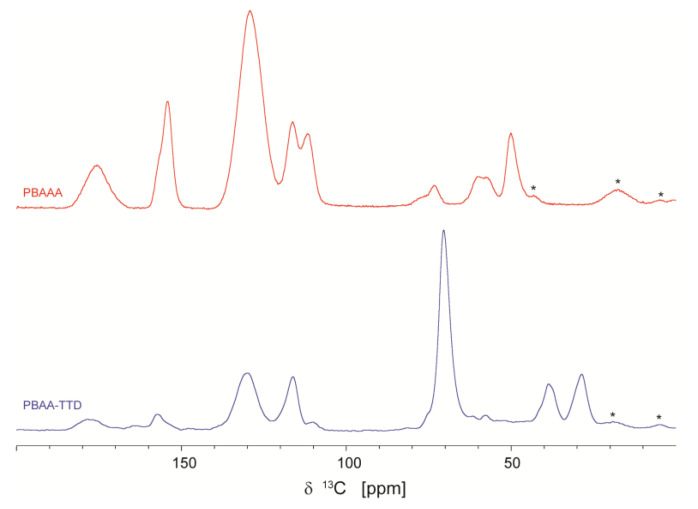
The ^13^C ss-NMR spectra of **PBAAA** (red) and **PBAA-TTD** (dark blue) recorded by the CP-MAS sequence as described in Section 3, * indicating the sidebands.

Figure 3 compares the ^13^C ss-spectra of the initial polymer **PBAAA** and the cross-linked polymer **PBAA-TTD**. The opening of lactone rings was confirmed by the disappearance of the peak at δ = 50 ppm and the drastic reduction of the peak at 110 ppm. Additionally, new peaks not found in **PBAA** appear in the ^13^C *ss*-NMR spectrum of the **PBAA-TTD** polymer at δ = 28 ppm, 38 ppm, and 70 ppm. They are attributed to the -CH_2_- groups of the diamine **TTD** used for cross-linking. Other peaks that demonstrate that the cross-linking reaction has occurred are those specific to the benzene ring modifying the aromatic region in the ^13^C ss-NMR spectrum, as mentioned above.

**Figure 4:**
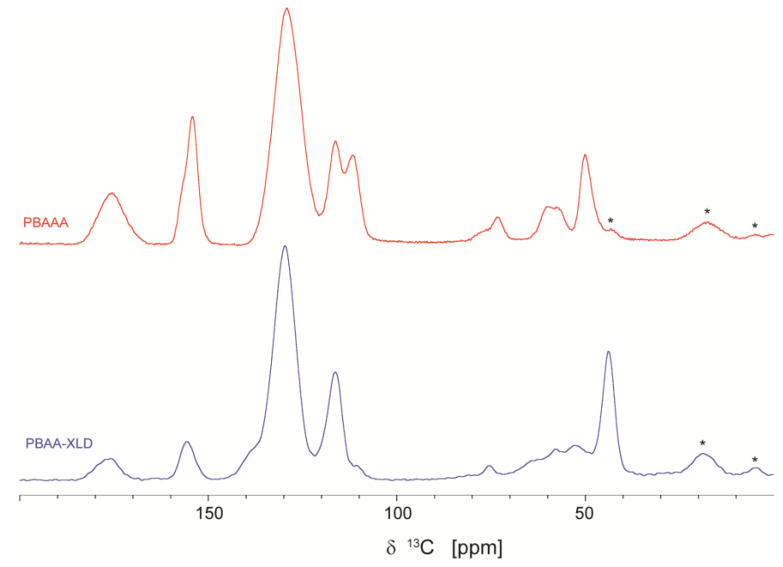
The ^13^C *ss*-NMR spectra of **PBAAA** (red) and **PBAA-XLD** (dark blue) recorded by the CP-MAS sequence as described in Section 3, * indicating the sidebands.

In the ^13^C ss-NMR spectrum of **PBAA-XLD**, the signal pertaining to the carbon atom of -CH_2_-Ph- from the cross-linking agent **XLD** (Figure 4) appears at 44 ppm. The signal attributed to the carbon -CH- atoms from the lactone ring of the polymer chain is considerably diminished, and it manifests as a shoulder at 50 ppm in the **PBAA-XLD** spectrum. The signals corresponding to the carbon atoms in the benzene rings, carboxylic and ester atoms, as well as those from the newly formed amide groups, are observed in the 110–180 ppm area. It is notable that, again, the signal at 111 ppm, typical for benzofuranone, diminished while an increase in intensity occurred at 116 ppm.

### 2.3. FTIR Spectroscopy of PBAAA and Their Cross-Linked Derivatives

**Figure 5:**
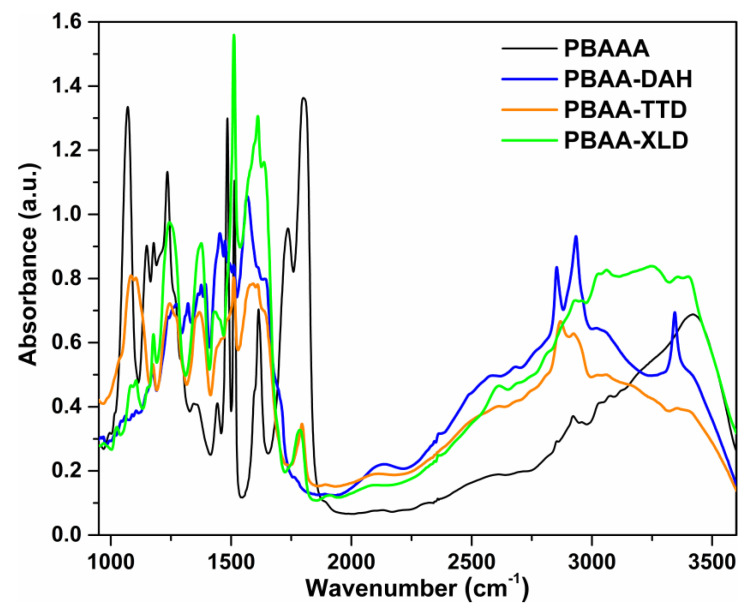
FTIR spectra of **PBAAA** and the cross-linked polymers **PBAA-DAH**, **PBAA-TTD** and **PBAA-XLD**.

The present study employs infrared spectroscopy to investigate the chemical properties of **PBAAA** and its cross-linked derivatives. This analysis aims to gain a deeper understanding of the molecular structure and the functional groups present in the materials. By examining the spectra, the chemical composition of the samples can be determined, and changes can be identified that may be attributed to the cross-linking process. The FTIR spectra of the **PBAAA** polymer and its cross-linked polymers **PBAAA-DAH**, **PBAAA-TTD**, and **PBAAA-XLD** are presented in Figure 5. The FTIR spectra were acquired using an equal amount of polymer, facilitating a rough quantitative and comparative analysis. Despite the use of diamines with varying reactivity during the cross-linking reaction, all the FTIR spectra demonstrate a discernible adsorption band at approximately 1800 cm^−1^, indicating the presence of the C=O bond in the lactone ring of benzofuran. However, this band appears diminished compared to **PBAAA**, indicating the opening of a portion of the lactone rings during the cross-linking reactions. Additionally, an increase in the intensity of the absorption band specific to the amide bond can be observed around the value of 1620 cm^−1^ in all cross-linked polymer spectra. The FTIR spectra of the cross-linked polymers **PBAA-DAH** and **PBAA-TTD** display high-intensity adsorption bands characteristic of the -CH_2_- group present in the cross-linking agent. These bands are observed at wave numbers 2929 and 2862 cm^−1^, which indicates the successful reaction of **PBAA** with the two diamines. However, these intense bands overlap with others in the FTIR spectrum of the PBAA-XLD cross-linked polymer.

### 2.4. Scanning Electron Microscopy

**Figure 6:**
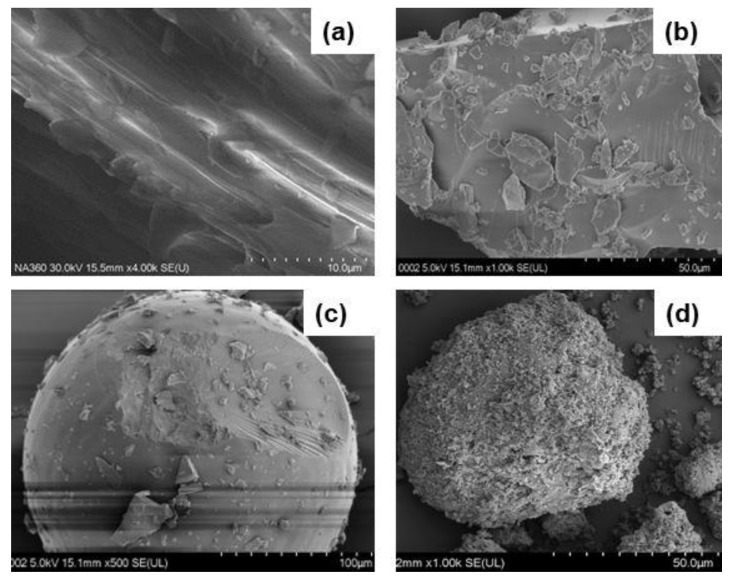
Scanning electron micrographic (SEM) images of (**a**) **PBAAA**; (**b**) **PBAA-DAH**; (**c**) **PBAA-TTD**; and (**d**) **PBAA-XLD**.

In general, the chemical structure of a cross-linking agent plays a crucial role in the final morphology of the polymer. The resulting morphology can be affected by several factors, such as the degree of cross-linking, the type and density of the cross-links, and the chemical composition of the polymer. SEM has been employed to investigate the morphological differences that arise in cross-linked polymers. In Figure 6, SEM images of the cross-linked polymers **PBAA-DAH**, **PBAA-TTD**, and **PBAA-XLD** are presented, along with the SEM image of the starting polymer **PBAAA** (Figure 6a), to facilitate comparative analysis. It is evident that the morphology of the cross-linked polymers exhibits significant variations depending on the type of linker. The starting polymer **PBAAA**, which resembles an arboreal self-assembling structure (Figure 6a), displays significant morphological differences compared to **PBAA-DAH**, which forms a compact block structure (Figure 6b). Juxtapose, the cross-linked polymers **PBAA-TTD** and **PBAA-XLD** that possess improved thermal conductivity (v. i.) form spherical structures (Figure 6c,d).

### 2.5. Thermogravimetric Measurements

**Figure 7:**
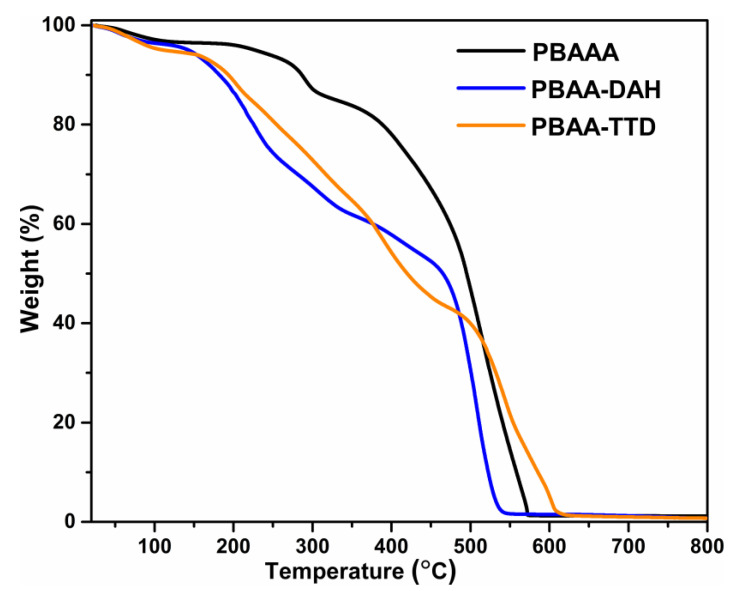
TGA thermograms obtained on the **PBAAA** and the cross-linked polymers **PBAA-DAH**, **PBAA-TTD** and **PBAA-XLD** from room temperature to 800 °C.

The thermal properties of polymers are susceptible to modification brought about by cross-linking. The TGA thermograms obtained with **PBAAA**, **PBAA-DAH**, and **PBAA-TTD** from room temperature to 800 °C are shown in Figure 7 and reveal that all polymers undergo decomposition in several steps, obviously because of the presence of different functional groups in their structures. The polymer **PBAAA**, in its initial form, exhibits a notable level of thermal stability up to a temperature of 300 °C. At higher temperatures, continuous decomposition occurs until the final state at 570 °C. The cross-linked polymers **PBAA-DAH** and **PBAA-TTD** start to decompose at lower temperatures. After evaporating water till 70 °C, they lose 6% mass up to 160 °C and undergo faster decomposition afterwards, showing several steps.

The TGA curve of **PBAA-DAH** showed a total mass loss of 49% from 160 °C to 450 °C. The final stage of decomposition occurred from 450 °C to 550 °C, leading to the mass loss of the remaining 45%. Conversely, the TGA curve of **PBAA-TTD** revealed three additional decomposition stages after heating the sample above 160 °C. During the first stage, from 160 °C to 376 °C, a mass loss of 34% was observed. In the second stage, from 376 °C to 490 °C, a mass loss of 19% occurred, while in the final stage, from 490 °C to 620 °C, a loss of 41% of the total mass was observed. These findings evidenced that the two cross-linked polymers suffer a more substantial mass loss until 540 °C than the starting **PBAAA,** thus exhibiting a different thermal decomposition behaviour. On the other hand, the cross-linked polymers’ stability until 160 °C can be sufficient for many applications.

In conclusion, the initial polymer shows enhanced thermal stability when compared to the cross-linked polymers. The proposed mechanism highlights that the degradation of **PBAAA-DAH** and **PBAAA-TTD** in an air atmosphere can be influenced by multiple factors, including chain scission and the breakdown of more accessible functional groups (such as the amine group). During thermal decomposition, free radicals are formed, which play an essential role in accelerating chain scission, contributing to the observed reduction in thermal stability of the cross-linked polymers.

### 2.6. Determination of Thermal Parameters

The thermal properties of the obtained samples were examined using the TPS method at three different temperatures, namely, room temperature (RT), 50 °C, and 100 °C. The TPS method is a reliable and well-established technique for analysing thermal properties. This investigation is expected to significantly contribute to the understanding of the characteristics of thermal management, which is crucial for various industrial and scientific applications. The performance of materials used in high-temperature applications depends on their thermal conductivity. When the goal is to minimise heat losses, low thermal conductivity values are required. Conversely, materials with higher thermal conductivities are preferred when heat transfer from one place to another is necessary. Therefore, having accurate and reliable thermal conductivity values is crucial when selecting a material for a specific application. This ensures that the material performs optimally in its intended use.

**Figure 8:**
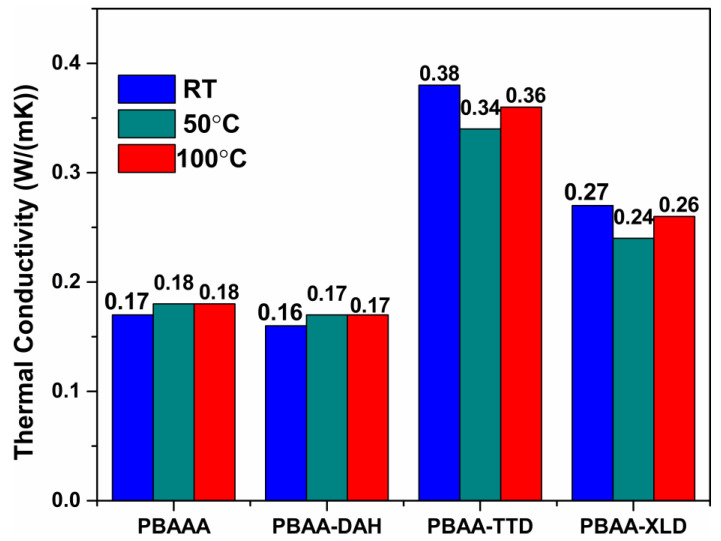
Thermal conductivity results for **PBAAA** and cross-linked samples of **PBAAA** with diamines at RT (blue column), 50 °C (green column), and 100 °C (red column).

Figure 8 compares the thermal conductivity values of the starting polymer and the cross-linked polymers. The results indicate that the structure of the cross-linking agent exerts an interesting influence on the thermal conductivity values of the polymer. When using the hydrophobic **DAH** diamine with its flexible alkyl chain as a cross-linking agent, the thermal conductivity values of the resulting **PBAA-DAH** are almost the same as those of the starting polymer **PBAA**, e.g., 0.16 W/(mK) versus 0.17 W/(mK) at room temperature, respectively. When using **PBAA-XLD**, where the cross-linking agent is an aromatic diamine with a stiff benzene ring, the thermal conductivity slightly increases from 0.17 W/(mK) to 0.27 W/(mK) at room temperature. The highest increase in thermal conductivity compared with the starting polymer is observed in the case of **PBAA-TTD** when a strongly polar diethylene glycol derivative is used as a cross-linker. The thermal conductivity increases by 124%, reaching the value of 0.38 W/(mK).

In a polymer chain, thermal conduction relies on the vibrations of molecules or atoms around fixed positions. When heat is applied, these vibrations transfer thermal energy to adjacent molecules. Phonons act as the main carriers of this energy. In the crystalline regions of polymers, closely connected atoms vibrate slightly around their equilibrium positions, enabling rapid heat transfer along the molecular chains. However, in the PBAAA case, the crystallinity degree is low due to the polydispersity of molecule weight. In addition, the anharmonic vibration of the molecule chains and crystal lattice, as well as defects of the boundaries of crystals, can lead to the scattering of phonons and affect phonon transport, resulting in low λ values.

To clarify the relationship between heat conduction and the cross-link polymer network, the structural change induced by cross-links will first be analysed. The formation of cross-link polymers has few effects. First, the cross-link can shrink the whole system by shortening the distance among molecules. The shrinkage of the polymer matrix can lead to the enhancement of the non-bond interaction, which can contribute to the heat transfer enhancement. In our case, the cross-link can also connect the polymer PBAAA chains and transform intermolecular van der Waals interactions into intramolecular covalent bonds, which connects heat conduction overall. Thus, the thermal conductivity will intuitively increase with cross-linked density due to the broad connection of heat pathways. Together, these structural factors promote the increase in thermal conductivity with cross-linked densities.

When polymers undergo cross-linking reactions, their thermal conductivity decreases with increasing temperature. This is an important handicap in practical applications when the polymer is part of a device that warms up during usage [26]. However, in the case of **PBAAA** and its cross-linked products, the thermal conductivity remains almost constant at elevated temperatures, a property making them promising for practical applications.

**Figure 9:**
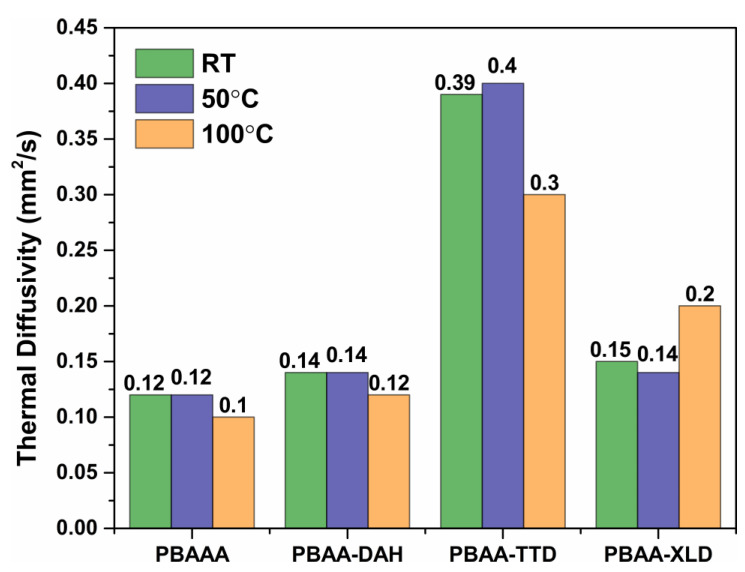
Thermal conductivity of **PBAAA** and cross-linked samples **PBAAA-DAH**, **PBAA-TTD**, and **PBAA-XLD** at RT (green), 50 °C (blue), and 100 °C (orange).

In addition to the thermal conductivity, the thermal diffusivity was measured (Figure 9), giving information about the rate of heat transport. Utilising **DAH** and **XLD** as cross-linking agents did not yield a substantial change; their thermal diffusivity values were nearly indistinguishable. Conversely, **TTD,** as a cross-linked agent, generated significant modifications. The thermal diffusivity was augmented by 225% from the initial polymer **PBAAA**, rising from 0.12 mm^2^/s to 0.39 mm^2^/s for **PBAA-TTD**. Again, the diethylene glycol diamine gave the best result.

An important issue in the fields of thermal conductivity and thermal diffusivity of materials is that both parameters remain stable when subjected to increased temperatures. Thus, these parameters can be considered reliable indicators of a material’s ability to distribute heat evenly. This target can be achieved in the case of **PBAAA** by cross-inking with appropriate diamines, particularly with the polar **TTD** (Figure 9).

## 3. Materials and Methods

### 3.1. Materials

The **PBAAA** synthesis was conducted following a previously reported procedure [27]. All reagents used were purchased from Merck (Darmstadt, Germany) and Alfa Aesar by ThermoFisher Scientific (Kandel, Germany) and were utilised without further purification.

#### Cross-Linking Reaction of PBAAA with Diamines

In a round-bottom flask, 2 g of **PBAAA** was dissolved in a mixture of 70 mL of methanol and 30 mL of deionised water. Subsequently, 1 g of diamine (1,6-diaminohexane (**DAH**), 4,7,10-trioxa-1,13-tridecanediamine (**TTD**), and p-xylylenediamine (**XLD**)) was added to the solution (Scheme 1). The mixture was subjected to ultrasonication for 1 h and refluxed under magnetic stirring for 12 h. The resulting solids were suspended in methanol. The precipitate was filtered off and washed several times with methanol and water to remove any unbound polymer and diamine. Finally, **PBAA-DAH**, **PBAA-TTD**, and **PBAA-XLD** products were dried in an oven at 60 °C and analysed.

### 3.2. Methods

A Jasco FTIR-6100 spectrophotometer (JASCO Deutschland GmbH, Pfungstadt, Germany) with a resolution of 0.5 cm^−1^ was used to record the infrared absorption spectra in the 400–4000 cm^−1^ range. Scanning electron microscopy analysis was performed using a Hitachi SU8230 High-Resolution Scanning Electron Microscope (Hitachi Ltd., Tokyo, Japan) equipped with a cold field emission gun. The samples were deposited on aluminium stubs and coated with a 10 nm gold layer for the morphological investigation. Thermogravimetric analysis was conducted using alumina pans in airflow at a rate of 50 mL/min within a temperature range of 30–800 °C, with a heating rate of 10 °C/min, utilising TA Instruments SDT Q 600 equipment (TA Instruments Inc., New Castle, DE, USA).

Solid-state Nuclear Magnetic Resonance ^13^C (ss-NMR ^13^C) spectra were recorded at 125.73 MHz Larmor frequency with a Bruker Advance III 500 MHz wide-bore NMR spectrometer operating at room temperature, using a 4 mm double resonance (1H/X) MAS probe. Standard Ramped-Amplitude Cross Polarisation in Magic-Angle-Spinning (RAMP CP-MAS) NMR spectra were acquired at 14 kHz spinning frequencies, 2 ms contact times and proton decoupling under a two-pulse phase-modulated (TPPM) sequence. The acquisition parameters were optimised to the following values of relaxation delay/number of averaged transients for specified investigated samples: 2 s/31,000(**PBAA**), 75 s/220 (**XLD**), 2 s/29,000 (**PBAA-DAH**), 3 s/14,000 (**PBAA-TTD**), and 2 s/31,000 (**PBAA-XLD**). For the **DAH** sample, the ^13^C ss-NMR spectrum was recorded using one-pulse (with proton High Power Decoupling), the 90° pulse length applied on the ^13^C channel being 4 µs, the decoupling field on the proton channel was 25 kHz, the relaxation delay was 3 s, and 48 transients were acquired. The recorded spectra are calibrated relative to the CH_3_ line in TMS (tetramethylsilane), through an indirect procedure that uses the α form of L-glycine as the external standard (C=O of glycine at 176.5 ppm). ^15^N CP-MAS NMR spectra of all samples were acquired at 7 kHz spinning frequency using a 4 mm double resonance (1H/X) MAS probe. The contact time for the standard CP-MAS pulse sequence was 4 ms for all experiments. The spectra were recorded under high-power proton decoupling (100 kHZ) using a TPPM (Two Pulse Phase Modulation) sequence, with a recycle delay of 2 s (**PBAA-DAH**, **PBAA-XLD**), 3 s (**PBAA-TTD**), 5 s (**DAH**), and 75 s (**XLD**). The number of averaged transients for investigated samples is as follows: 91k (**PBAA-DAH**), 51,000 (**PBAA-TTD**), 200,000 (**PBAA-XLD**), 110,000 (**DAH**), 670 (**XLD**). These spectra were calibrated relative to nitromethane using glycine (−347.6 ppm) as an external standard.

The thermal conductivity and diffusivity were measured in a Hot Disk TPS 2500S (Hot Disk AB, Kagaku, Sweden) apparatus with a 5464F1 sensor using the transient plane source (TPS) method. The method’s principle consists of applying a short heat pulse of a predetermined duration to the sample, initially kept at thermal equilibrium using a TPS sensor with a double function: a constant heat source and temperature sensor placed between two identical samples. The transient temperature response of the samples is recorded and further used to estimate the thermal conductivity. To get results with excellent accuracy, the samples were prepared in the form of identical pellets with a radius of 5 cm and a thickness of about 4 mm to ensure that we could use a TPS sensor with a diameter of 2 mm so that the heat generated by the spiral area does not diffuse to the sample outside boundary within a predefined period of measurement time.

## 4. Conclusions

We have demonstrated that reactions of a phenoplast-related PBAAA with diamines occurred at the lactone groups of benzofuranone by ring opening and at carboxylic acid moieties by condensation and led to cross-linkage. In addition, amide formation can also take place with just one amino group of the diamine, leaving unreacted the other one. Thus the products show a zwitterionic character comprising ammonium and carboxylate groups. Cross-linking of **PBAAA** with diamines allows the effect of the thermal properties, i.e., providing high values of thermal conductivity and diffusivity. It turned out that the properties of the cross-linking diamine, such as hydrophilicity and structural rigidity, are important factors for achieving the prospective properties of the polymers, rendering them promising candidates for various applications. Cases were found wherein the thermal conductivity did not drop with rising temperature, a rare property important for practical applications in electronic devices, aerospace, and automotive manufacturing or heat exchangers.

## Data Availability

Data are contained within the article and Supplementary materials.

## Supplementary Materials

The following supporting information can be downloaded at: https://www.mdpi.com/article/10.3390/molecules29246020/s1, Figure S1. Photos of starting polymer **PBAAA** and cross-linked polymer **PBAAA-DAH**, **PBAAA-TTD** and **PBAAA-XLD**; Figure S2. The ^15^N ss-NMR spectra of **PBAA-TTD** recorded by the CP-MAS; Figure S3. The ^15^N ss-NMR spectra of **PBAA-XLD** and **XLD** recorded by the CP-MAS.
