# Supplementary material for: Reaction of Lactone-Containing Poly(benzofuran-co-arylacetic acid) with Diamines to Cross-Linked Products of Improved Thermal Conductivity
Source: Molecules. 2024 Dec 20;29(24):6020. doi: 10.3390/molecules29246020 (PMC11676040; doi:10.3390/molecules29246020)
Supplement: Supplementary file 1 [file molecules-29-06020-s001.zip › molecules-3359294-supplementary.pdf]

molecules

MDPI

Article

Reaction of Lactone-Containing Poly(benzofuran-*co*-arylacetic acid) with Diamines to Cross-Linked Products of Improved Thermal Conductivity

Alexandrina Nan [1], Xenia Filip [1] and Jürgen Liebscher [1,2,*]

[1] National Institute for Research and Development of Isotopic and Molecular Technologies, Str. Donat 67-103, 400293 Cluj-Napoca, Romania; alexandrina.nan@itim-cj.ro (A.N.); filip.xenia@itim-cj.ro (X.F.)
[2] Institute of Chemistry, Humboldt-University of Berlin, 12489 Berlin, Germany
* Correspondence: liebscher@chemie.hu-berlin.de

Supplementary Materials

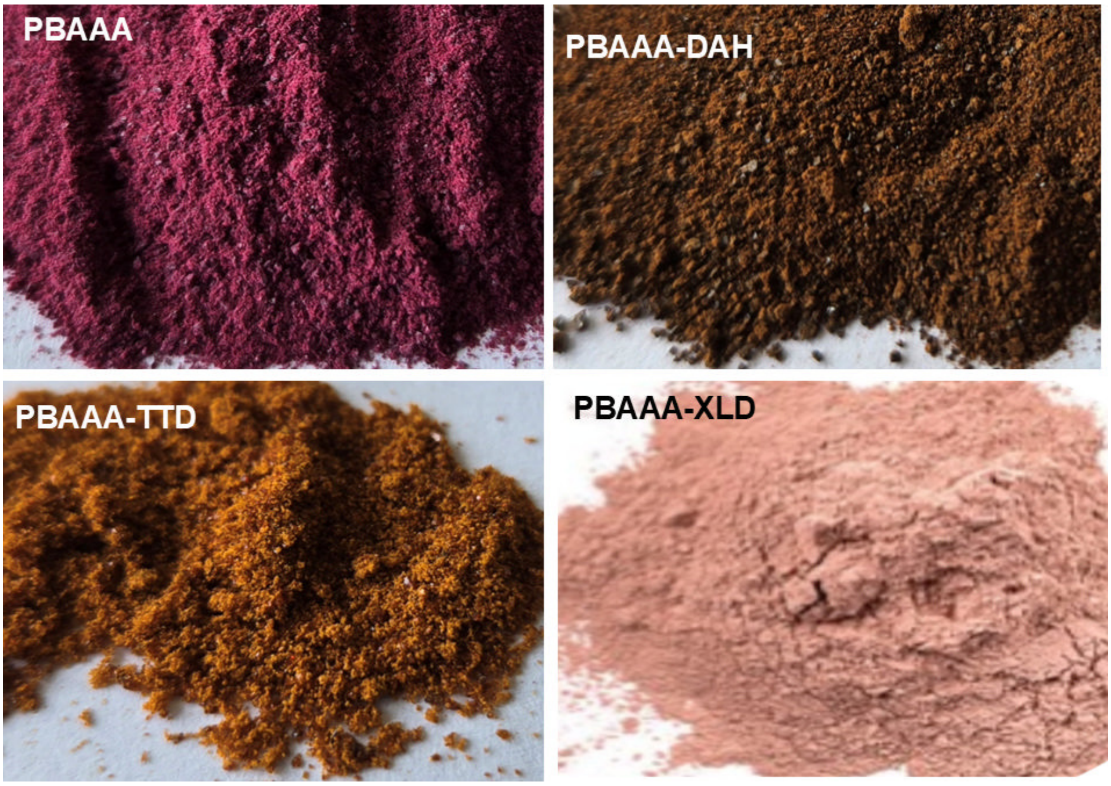

Figure S1. Photos of starting polymer **PBAAA** and crosslinked polymer **PBAAA-DAH**, **PBAAA-TTD** and **PBAAA-XLD**

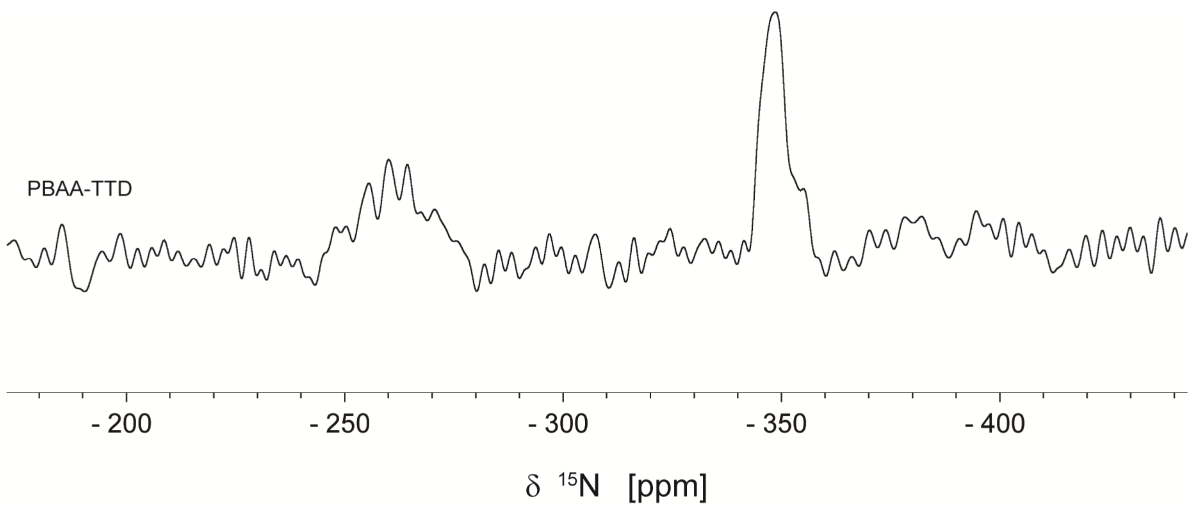

Figure S2. The ^{15}N ss-NMR spectra of **PBAA-TTD** recorded by the CP-MAS

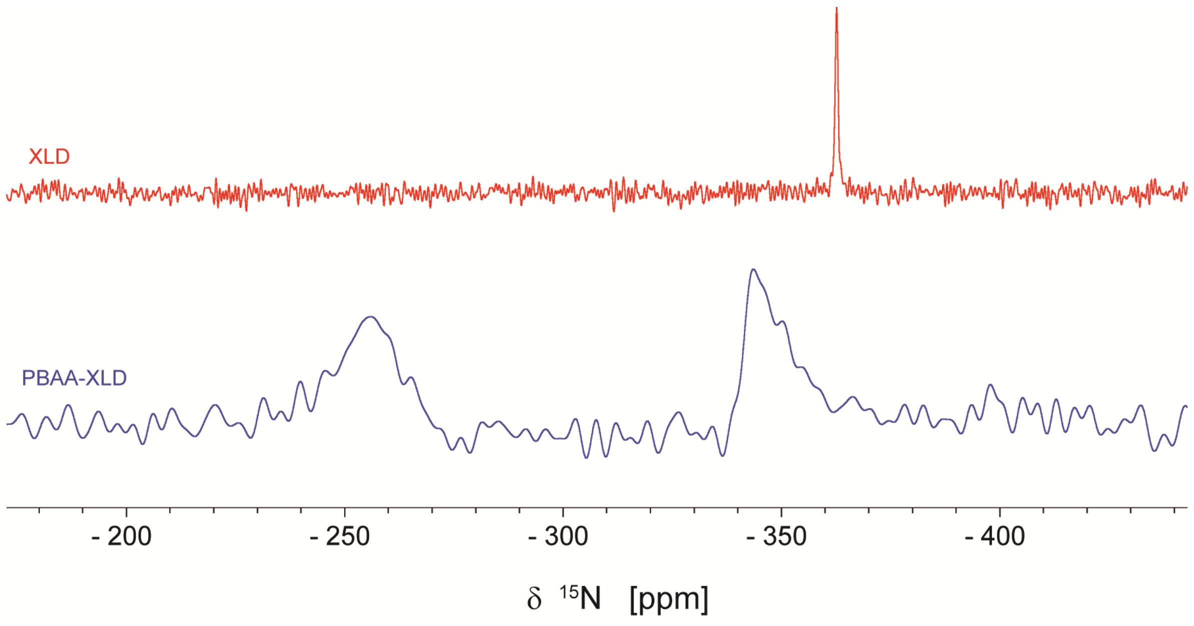

Figure S3. The 15N ss-NMR spectra of PBAA-XLD and XLD recorded by the CP-MAS
